# WHEN I’M 64 – LONGITUDINAL OBSERVATIONS FROM AN INTERDISCIPLINARY COURSE ON AGING FOR FIRST-YEAR STUDENTS

**DOI:** 10.1093/geroni/igae098.3769

**Published:** 2024-12-31

**Authors:** Karuna Thomas, Matthew Picchiello, Nancy Morrow-Howell, Susan Stark, Brian Carpenter

**Affiliations:** Washington University in St. Louis, St. Louis, Missouri, United States; Washington University in St. Louis, St. Louis, Missouri, United States; Washington University in St. Louis, St. Louis, Missouri, United States; Washington University in St. Louis, St. Louis, Missouri, United States; Washington University in St. Louis, St. Louis, Missouri, United States

## Abstract

Research has highlighted the value of education for college students on the aging process. Curricula have the potential to address misconceptions about aging and draw students toward further coursework and experiences with older adults. This study explored the impact of participation in an interdisciplinary course on aging across one year for the Fall 2023 cohort (N = 50) with a control group that did not participate in the course (N = 33), and after five years for the Fall 2017 cohort (N = 59) and control group (N = 57). The course was offered to first-year undergraduate students. Weekly classes included older adults from the community. Surveys assessing attitudes towards aging and participation in aging-related activities were administered prior to and at the end of the academic year, and an annual follow-up. Group-by-time analyses showed that participants from the class had significantly more positive attitudes towards aging [F(1, 82) = 13.67, p <.05, ηp2 =.03], and significantly fewer ageist beliefs [F(1, 82) = 9.21, p <.05, ηp2 =.02] at the end of the academic year compared to the control group. In the 5-year follow-up, students in the course also reported more engagement in aging-related fields compared to the control group [t(114) = 4.65, p <.05]. The course content was reported to be relevant to 45.8% of former students in their professional life, and 57.6% in their personal lives. These results substantiate the short and long-term positive impact of engaging undergraduate students in curriculum on aging.

